# Cold Powder

**Published:** 1875-01

**Authors:** 


Cold Powder.—We have long been in the habit of using what we call a *cold powder*, which we have found of great value in breaking up colds when taken in time, and in modifying their force when taken late.

The prescription is as follows:

Camphor, five parts. Dissolve in ether to the consistence of cream. Then add carbonate of ammonia, four parts; opium powder, one part.

Mix and keep in a tightly corked bottle. The dose is of course regulated by the opium, and ranges between three and ten or fifteen grains. We have been accustomed to prescribe it for our friends by the finger-nail full, or as much as one can put on the finger-nail.

This powder may be taken in a little water just before retiring, by preference, or at any hour of the day, whenever there is a *suspicion of having caught cold.* If need be, a moderate dose may be taken several days in succession.

The advantages of this powder are very great:

1. The taste is agreeable, or, at least, is not disagreeable. Even the bitterness of the opium is mostly neutralized by the camphor and ammonia. No child objects to this powder.

2. It is singularly and inexplicably efficacious. We believe it to be more efficient than Dover's powder, and incomparably more agreeable. In some cases it produces a gentle perspiration; in others, this special effect is not observed. It is so easy to take, and so harmless in small doses, that it is well and safe to take it whenever we become badly chilled.—*Archives of Electrology and Neurology.*

